# Perceptions of newly admitted undergraduate medical students on experiential training on community placements and working in rural areas of Uganda

**DOI:** 10.1186/1472-6920-10-47

**Published:** 2010-06-23

**Authors:** Dan K Kaye, Andrew Mwanika, Patrick Sekimpi, Joshua Tugumisirize, Nelson Sewankambo

**Affiliations:** 1 Makerere University College of Health Sciences, P.O. Box 7072, Kampala, Uganda

## Abstract

**Background:**

Uganda has an acute problem of inadequate human resources partly due to health professionals' unwillingness to work in a rural environment. One strategy to address this problem is to arrange health professional training in rural environments through community placements. Makerere University College of Health Sciences changed training of medical students from the traditional curriculum to a problem-based learning (PBL) curriculum in 2003. This curriculum is based on the SPICES model (student-centered, problem-based, integrated, community-based and services oriented). During their first academic year, students undergo orientation on key areas of community-based education, after which they are sent in interdisciplinary teams for community placements. The objective was to assess first year students' perceptions on experiential training through community placements and factors that might influence their willingness to work in rural health facilities after completion of their training.

**Methods:**

The survey was conducted among 107 newly admitted first year students on the medical, nursing, pharmacy and medical radiography program students, using in-depth interview and open-ended self-administered questionnaires on their first day at the college, from October 28-30, 2008. Data was collected on socio-demographic characteristics, motivation for choosing a medical career, prior exposure to rural health facilities, willingness to have part of their training in rural areas and factors that would influence the decision to work in rural areas.

**Results:**

Over 75% completed their high school from urban areas. The majority had minimal exposure to rural health facilities, yet this is where most of them will eventually have to work. Over 75% of the newly admitted students were willing to have their training from a rural area. Perceived factors that might influence retention in rural areas include the local context of work environment, support from family and friends, availability of continuing professional training for career development and support of co-workers and the community.

**Conclusion:**

Many first year students at Makerere University have limited exposure to health facilities in rural areas and have concerns about eventually working there.

## Background

In Uganda, as in many low-income countries, there is an acute problem of adequate human resources especially in rural areas. There is absence of the necessary skills mix, a problem associated with geographical mal-distribution of health professionals and difficulty in deploying or retaining them in some areas of the countries [1]. There is a critical shortage of doctors and other health professionals in rural areas of Uganda, partly due to health professionals' unwillingness to work in a rural environment or certain other areas [2]. One crucial strategy to address this problem is to arrange health professional to have experience in rural environments through rural placements so that they acquire experience of health systems and health services in rural areas part as part of their training [3]. The predictors of health professionals' choices for recruitment or retention on jobs in rural areas include the individual having been born or educated in a rural location, exposure to rural healthcare during training and access to continuing professional education [4]. Others are good relationships with peers and colleagues, ability to adapt to or adopt the rural 'lifestyle', successful integration into or interaction with local communities and family and spousal contentedness [4]. It is not well documented whether, how and why these factors apply to the Ugandan context.

Makerere University Faculty of Medicine changed training of medicine and nursing students from the traditional curriculum to a problem-based learning curriculum in 2003. The first graduates of nursing and medicine on this curriculum completed their studies in 2007 and 2008 respectively. The Faculty of Medicine carried out a comprehensive evaluation of the problem-based learning (PBL) curriculum to assess the likely influence of the graduates' training experience on willingness to be recruited to work in rural health facilities. Continuing medical students (second, third, fourth and fifth years) had already received orientation on the role of community-based education, had been informed about importance of community-based training irrespective of medical discipline, and had already been involved in at least one community placement.

Over 80% of the Ugandan population resides in the rural areas. There are several educational benefits of rural placements for health professional training. Students acquire knowledge and skills in real-life contexts of ill-health and its related non-biomedical determinants. By enabling students to learn for significant periods in rural communities, health professional training institutions can address the medical workforce needs of the communities they serve at the same time as providing educational advantages to their students [5]. It is therefore necessary to address characteristics that would influence students' working in rural areas on qualification [6]. Addressing these factors during medical training, especially during community based training, may increase recruitment, deployment or retention of health workers in the rural areas. The objective was to assess first year students' perceptions on experiential training through community placements and factors that might influence their willingness to eventually work in rural health facilities after completion of their training.

## Methods

The assessment was part of the comprehensive evaluation exercise for the PBL curriculum that was conducted from 19^th^-31^st ^October 2008 at Makerere University College of Health Sciences, Kampala, Uganda.

### Participants and data collection

The survey was conducted among 107 of the 120 newly admitted first year medical students on the medicine (76 of 84), dentistry (8 of 8), nursing (8 of 10), pharmacy (10 of 12) and medical radiography (5 of 6) program students. An open-ended self-administered questionnaire (developed by the staff of the college of health sciences and external consultants who were involved in the evaluation of the PBL curriculum) was given to first year students on their first day at the College of Health Sciences, during their first meeting with the college administration. Data was collected on sex, age, district of origin, and district where the immediate former school was located. The specific questions asked were: What 2 two factors motivated you to apply for a program in the medical faculty? Did you get career guidance before making your choice? Do you have a mentor (someone senior health professional who guided you in making the choices)? In case you have a mentor, what 2 (two) roles did the mentor play in your choice and what role do you expect your mentor to play in your training? Have you been exposed to rural health facilities? If Yes, in what roles have you been exposed to the facilities? What was your experience of health care providers in rural health facilities? What are your experiences of urban health facilities? What factors would you consider before making your choices? Would you be willing to work in rural areas at completion of your training? What are your views about having part of your training conducted within the rural communities?

Data was therefore collected on motivational factors for the students to apply for a program in medical field, whether the newly admitted students received career guidance before making their choice, whether they have mentors and the roles two primary roles of the mentors in their training. Data was also collected on prior exposure to rural health facilities and capacity in which they were exposed (patient, healthcare provider, patient caretaker), the students' perception/experience of healthcare providers in rural health facilities, prior perception and/or experiences of urban health facilities. Other data collected was on factors they would consider before making a choice to work in a rural area, and their views on having part of your training conducted within the rural communities.

To gain a deeper understanding of the students' vies on community-based training and willingness to work in rural areas at completion of the training, in depth interviews were conducted by one of the authors (DKK) assisted by a research assistant, using a standardized interview guide. The participants were purposively selected by maximum variation sampling so as to represent views of the upgrading students, students from homes in urban or very rural areas, students with and those with no prior experience in rural health facilities, and students with very negative or very positive views on community-based training. The issues explored were motivation for career choice in the medical field, prior exposure to health facilities in rural areas, perceptions or experiences of health facilities in rural areas, factors that would influence their choices to work in rural areas, and their vies on having part of the medical training conducted at rural health facilities.

### Data analysis

For data analysis, the open-ended answers, which were initially entered in Microsoft Excel Software, were summarized into related thematic areas, post-coded and presented as frequencies and percentages. Qualitative data analysis involved development of codes and categories (expressions or phrases with similar meanings) by DKK and two research assistants, according to key concepts from the transcripts. Systematic comparison and re-classification of emerging codes and categories across texts was done using the method of latent content analysis. Easy Text (EZ) software was used for data retrieval.

### Ethical considerations

This survey received ethical approval from the Research and Ethics Committee of Makerere University Faculty of Medicine. All the participants gave informed consent to participate, with guarantees that the information they provided was confidential and there were no personal identifiers in any documents or subsequent reports.

## Results

Of the 107 students, the mean age of participants was 20.4 (± 2.8) years; 76 participants (69.7%) were male. Whereas only about one third had their home district in the central region, over 3 out of 4 completed their high school from schools located in the central region, and possibly were not conversant with rural areas. Table 1 shows the districts of origin or birth and the districts in which the students completed their secondary school training. Whereas only about one third had their home district in the central region, over 3 out of 4 completed their high school from schools located in the central region which are more urbanized or peri-urban, and possibly were not conversant with rural areas. Table 2 shows the primary and secondary reasons which acted as motivation for choosing a career in the medical field as well as whether the students had mentorship. The main reasons were high disease burden, few doctors are available or desire to improve health standard, desire to save lives and desire to serve others or help others. Ninety three participants received career guidance and 63 had mentors. Table 3 indicates prior exposure of the participants to rural health facilities, the capacity or context in which participant got the exposure, overall perception of the rural health facilities and exposure to urban health facilities. Most first year students had minimal exposure to rural health facilities, yet this is where most of them will eventually have to work. Over seventy five percent of the newly admitted students were willing to have their training from a rural area.

**Table 1:** District of origin or home district and district where school is located

Region of Uganda	Districts of origin	Number and Percentage
Eastern (mainly rural)	Jinja, Mbale, Kamuli, Bugiri, Budaka, Pallisa, Kumi, Soroti, Mbale or Amuria)	22 (20.2%)

Western (mainly rural)	Kyenjojo, Masindi, Mubende, Hoima, Masindi, Mbarara, Bushenyi, Ntungamo or Kasese.	22 (20.2%)

Central (mainly peri-urban areas)	Kiboga, Mityana, Wakiso, Mukono, Kayunga, Mpigi and Kampala;	36 (33.0%)

Northern (mainly rural)	Lira, Dokolo, Amuru, Kitgum and Gulu	9 (8.3%)

South Western (mainly rural)	Kabale, Kisoro, Rukungiri	3 (2.8%)

Southern	Masaka, Rakai, Kalisizo	15 (13.8%)

**Region**	**Districts where former (high) school is located**	**Number and percentage**

Eastern(mainly rural)	Jinja, Mbale, Kamuli, Bugiri, Budaka, Pallisa, Kumi, Soroti, Mbale or Amuria)	6 (5.5%)

Western (mainly rural)	Kyenjojo, Masindi, Mubende, Hoima, Masindi, Mbarara, Bushenyi, Ntungamo or Kasese.	12 (11.0%)

Central	Kiboga, Mityana, Wakiso, Mukono, Kayunga, Mpigi and Kampala;	84 (77.1%)

Northern (mainly rural)	Lira, Dokolo, Amuru, Kitgum and Gulu	1 (0.9%)

Southern (mainly rural)	Masaka, Rakai, Kalisizo	4 (3.7%)

**Table 2:** The primary and secondary motivation for choosing a career in the medical field

Reason	Number and percentage of respondents
**Primary reason**	
To save lives	22 (20.2)
Desire to serve others or help others	21 (19.3)
Fulfilling a childhood dream	24 (22.0)
Prestige	7 (6.4)
Money, attractive salary or job security	4 (3.7)
High disease burden, few doctors are available or desire to improve health standard	22 (20.2)
None given	9 (8.3)

**Secondary reason**	
To save lives or help others	37 (33.9)
Prestige or attractive salary	17 (15.6)
Few health workers or high disease burden	9 (8.3)
Curiosity, desire to do research on humans or fulfilling a childhood dream	10 (9.2)
None given	34 (33.0)

**Career guidance and mentorship for trainees**	
***Received career guidance***	
Yes	93 (85.3)
No	14 (12.8)

***Has a mentor***	
Yes	63 (57.8)
No	36 (33.0)
No response	10 (9.2)

***Primary mentor role (n = 107)***	
Inspires	6 (5.5)
Supports materially or financially	17 (15.6)
Offers guidance or advice	28 (25.7)
Acts as a role model	3 (2.8)
No role given	55 (50.5)

***Secondary role (n = 107)***	
Motivates, encourages or advises	31 (28.4)
Financial or material support	13 (11.9)
Role model	3 (2.8)

**Table 3:** Exposure of participant to rural health facilities, the capacity or context in which participant got the exposure, overall perception of the rural health facilities and exposure to urban health facilities

Exposure to rural health facilities	Number and percentage
*Has ever been exposed to rural health facilities*	
Yes	48 (44.0)
No	48 (44.0)
No response	11 (11.9)

*Role or context of this experience or exposure (n = 48)*	
As patient	24 (50.0)
As healthcare provider	7 (14.6)
As patient caretaker	5 (10.4)
Patient caretaker or patient	12 (25.0)

*Other context in which you were exposed to rural health facility other than related to healthcare seeking*	
Visitor to the facility	12 (11.0)
Non-medical service provision (support supervision, counselor, planning visit, auditor)	7 (6.4)

*Perception of rural health facilities (n = 107)*	
Understaffing	10 (9.2)
Poor service or poor facilities	35 (32.1)
Health workers are rude, absent, have poor ethics	11 (10.1)
No comment	53 (48.6)

*Participants with prior exposure to urban facilities*	13 (11.9)

*Ready for training in a rural area*	80 (74.7) 27 (25.3)

Table 4 indicates the considerations by participants in making a choice to work in a rural health facility. Many first year students have concerns about working in rural areas. The decision to work in a rural area after qualification is likely to be facilitated by prior exposure to rural practice. This prior exposure may be attained during training through community-based education. Rural exposure provides understanding of the rural context. Factors that might influence retention include the local context and nature of work environment, role of family and friends, ongoing continuing professional training and career development, understaffing and quality of health service management, teamwork and support of co-workers as well as personal motivation which is reinforced by a positive relationship with the community.

**Table 4:** Considerations by participants in making a choice to work in a rural health facility

Reason	Possible considerations	Number and percentage
Security	Area is politically stable, personal safety, isolation and remoteness of the location of health facility	33 (30.3)

Workload	Presence of other staff in adequate numbers and professional mix, doctor-patient ratio, understaffing (support from other health workers)	22 (20.2)

Social amenities	Communication, transport, family, entertainment, language, ease of communication with patients, cost of living, salary and benefits, the culture of the local population, opportunity for recreation, schools for children, opportunity to earn extra income	24 (22.1)

Facilities at the health units	Equipment, infrastructure, opportunities for continuing education,	30 (27.4)

### Perceptions on community-based education

Many of the students gave positive views of community-based education and were in favor of this being part of their medical training. Some of the reasons given for its importance by the majority of students were that it enables students understand the medical conditions in rural areas, to see a variety of medical conditions (some of which are not seen in the teaching hospital), and to learn about the management of the health care system. This is exemplified by the view of one male student:

*'Some health units have basic facilities for teaching and some health workers there are willing to teach. Unlike in a large hospital, students in district hospital may get more hands-on experience during their training. Many of the units, especially Non-Government hospitals, have modest facilities for teaching, accommodation and student welfare. You can easily interact with staff and know how hospitals are run'*.

Other reasons in favor of community-based education, given by a few interviewees, were being able to explore different communities, providing relaxation from busy town life and opportunity for adventure. Students who gave negative views on community-based training expressed worries about the absence of necessities, being cut off from friends and colleagues, absence of guidance from faculty or any tutors and inadequate exposure to the variety of conditions, which exist in the large teaching hospital. Others were inadequate support facilities like internet and libraries to enable self-directed learning, and inability to understand the local languages or cultures.

### Perceived reasons for accepting posting to work in rural areas

One common theme as to why the newly admitted students would be unwilling to work in rural areas related to the poor socio-economic condition of the health workers in rural areas. Most interviewees suggested that they would only work in rural areas if the financial situation of the health workers in rural areas were to improve through active improvement of remuneration from the either the central government or the local government. The majority view was that current disparities in salaries for health workers in rural and urban areas and well as those in public and private sectors, even in the same locality, irrespective of whether this was rural or urban, would be a major consideration for them to choose which institution to work for. One respondent mentioned that the higher salaries of private sector employees attracted health workers to rural areas if this was where the posting station was available, giving the example of research projects that recruit and retain staff in rural areas. Such incentives would therefore attract health workers to rural areas.

Another area that needs to be addressed urgently to attract young graduates to rural areas is the style of leadership and management of health units in rural areas. The respondents that had exposure to rural health facilities thought the management were arrogant, inconsiderate and aloof to the plight of young staff. The health unit administration were considered unsupportive and it was felt they did not treat rural junior health professionals well. Personnel and staff welfare departments were identified as a specific problem area as most of the people in them were not of the medical background. Those who had no prior exposure to rural health facilities thought that the administration probably was ambivalent and rigid with poor leadership skills, which may explain the understaffing and related deterioration in rural services.

Whereas some students felt that money was the most important factor to retain them in rural hospitals, others stated that issues other than money that would give them job satisfaction were more important. These included availability of supplies and basic equipment, recognition of their plight by unit administrators and adequate protection at the workplace. Most students felt that the current working conditions in many rural areas were not conducive and contributed to the 'stress' of working in rural hospitals. A number of doctors stated that the working conditions were one of the most important factors contributing to good job satisfaction. One respondent exemplifies this view:

'I think the most important thing which has to be emphasized is that money is not the most important factor for any clinician to be working in a rural setting. Many are willing if an opportunity is available. However, the working environment has to be addressed. What is more important is job satisfaction: Is there equipment? Does the community or colleagues recognize your contribution? Does the unit have adequate supplies? Do you have colleagues to work with? At least there has to be a basic functional laboratory. Are there facilities like theatre, emergency resuscitation equipment or facilities for patient referral?'

Another reason that would be considered other than the physical infrastructure of the rural health units is the availability of basic communication facilities like telephone, internet connectivity, television access, availability of access roads with safe transport on the way to the facilities, as indicated by a female respondent:

'There must be basic infrastructure for accommodation, adequate access roads and telephone connections. There must be access to the market and leisure places for one to relax in after work. Without these, I would quickly get tired of the place and leave so I would rather not go there in the first place.'

The impact of lack of accommodation on recruitment or retaining doctors health professions in rural areas was given by most of the respondents. It was mentioned that many health workers who are already married may not be eager to work in rural areas without accommodation. Likewise, many doctors leave rural areas as soon as they want to start a family, and good accommodation mentioned as a major incentive especially for newly qualified health workers who want to settle quickly and so choose to work at a particular hospital, as exemplified by one respondent:

*Good basic accommodation facilities are very crucial. If one has to struggle with looking for accommodation at a facility where they are posted, or if one considers accommodation facilities inadequate, such as sharing bathrooms and toilet facilities with others, or lack of privacy, he/she will not enjoy the work environment and will leave as soon as possible*.

Opportunity for continuing health professional training was also cited as a major consideration for the choice of where one will eventually work or settle. Some areas were identified as not academically stimulating, where one 'gives without getting anything in return', where many health professionals may just 'rot'. The majority of respondents suggested that short courses for rural health professionals should be provided by the Ministry of Health or district health offices, and internet access facilities for the purposes of distance education should be provided to the health professionals that volunteer to work in rural areas. One student who was upgrading from being a laboratory technician to a medical doctor exemplifies this view:

*There is serious lack of academic stimulation in rural hospitals. You only work with the knowledge that you acquired during training. Many young health professionals are eager and willing to upgrade or improve their performance through continuing education, but opportunities are few and limited. Even opportunities for career progression are non-existent. Many young people may even want to update or improve their knowledge and skills, but opportunities are limited*.

Many respondents felt that training in rural areas would not be a problem if there were facilities provided at the rural areas, and more so if the lecturers were willing to guide and supervise them during their stay. The majority view was that learning from different environments may expose students to varied clinical situations which they may not see in a specialist teaching hospital. It was also noted that the quality of the rural health workers may improve if they get supervision from consultants, something that many respondents felt was necessary but probably lacking at the moment. Support from surgical specialty consultants was thought as more appropriate. However for rural training to succeed, respondents thought teachers must be committed to the program, as indicated by one respondent:

'Unless the teachers also come to the rural areas to supervise and teach in the field, the rural training would not be objective. It should also be clear what aspect you go their to learn. This period should be supervised or assessed so that some students do not view it as a waste of time or a holiday.'

Another perceived benefit of training in rural areas was early interaction with the community so that students would learn more about the different communities that have different cultures and lifestyle from theirs. This will build or strengthen relationships with the community, as exemplified by one interviewee:

*'The relationship between staff in the health units and the community is very important. Therefore, it is necessary for students to learn how to interact with the community and build relationships before they graduate and start working. If they can work without friction with the community, health professionals can work anywhere. But when there is interference, they just move on'*.

Factors that made health professions hate working in rural areas included insufficient salary, heavy work load and understaffing, poor housing, poor hospital management, lack of basic medical equipment, personal relationships, no recreational facilities and inadequate opportunities for continuing professional development, graduate training or promotion. On what interventions that might be instituted to attract and retain health professionals in rural areas, the suggestions included providing a hardship allowance for health professionals who sacrifice to work in rural areas. This could be in cash or kind such as subsidies on housing, telephones, electricity, and schooling for children. Other interventions may include offer of opportunities for postgraduate training, earlier consideration for promotion and provision of recreational facilities.

## Discussion

The study shows that first year health professional students at Makerere University have limited exposure to health facilities in rural areas and have concerns about eventually working in rural areas, which are related to personal factors, health system factors, socio-economic factors and prior exposure to rural health facilities. However, despite the importance of salaries, many doctors stated that other factors, such as job satisfaction and working conditions, were more important and the salary on its own would not retain them. Improvement in remuneration is one of the most important perceived factors in retaining health professionals in rural practice. Around the world, dissatisfaction with income is one of the major causes of doctors leaving public service [7], and improving salaries is often mentioned as an intervention to attract and retain rural doctors [8].

The decision to accept deployment to work and maintain jobs in rural areas is facilitated by exposure to rural practice during training, an understanding of rural needs and exposure to rural role models [9,10]. This can be achieved through conducting experiential training of health professionals at community training sites where they get exposure to the community in which they will eventually work. Research from Australia shows that students from a rural background were more willing to be trained or to work as doctors in rural areas [9]. This makes it vital to assess exposure of medical trainees to rural health facilities. It is also necessary to include community placements as a crucial aspect of the medical training programme, to identify potential barriers to the community-based training as well as factors that may impede eventual deployment and retention in rural areas. Since the context and nature of work and the environment influence the decision to remain in rural areas, there is need to orientate medical trainees to the future working environment as well as the health services, which students will meet upon qualification. The community placement ensures that individual motivation is reinforced by positive and supportive relationships with the community, which shapes and changes trainees values related to working in rural areas [11]. The situation of increasing inequities in access to healthcare calls for a search for innovative ways to improve the training, recruitment, deployment and attraction as well as retention of staff in remote areas. One strategy to address this problem is to assess and address factors which influence decisions to accept and/or stay in rural postings.

Community placement for health professional training regardless of discipline, enhances students' achievement of adaptation and participation in change, application of problem solving in new and future situations with the community serving as a real-life context, creative and critical thinking and adoption of holistic approach to problems and situations. As the students interact with different communities and different providers in the community, the students acquire appreciation of diverse viewpoints. Working in small groups at community training sites encourages successful team collaboration, trains students in leadership skills and optimizes utilization of relevant and varied resources. In a program on community placement in Australia [12], the rural internship provides medical students with valuable experience through active participation in the healthcare team, enjoying the social and cultural aspects of their attachment, the opportunity to further their understanding of rural communities. They report a rich clinical experience where they undergo limited supervised responsibility and acquire ability and confidence to undertake the role of the intern.

The critical shortage of doctors in rural areas is a strong motivation for providing rural experiences for medical students [3]. Rural-based training placements- might enable trainees to overcome the cultural shock of those who have never been to other areas of the country, or to rural areas [13,14]. Using a community-based training model to foster and prepare students to work in rural areas has been tried in Canada and the United States. In Canada and the United States, government-funding initiatives targeted at rural medical education through community placements plays an important role in preparing medical trainees for work in rural areas [15]. Though what motivates health workers to choose to work in rural areas are a complex interaction of personal, situational and environmental factors of the context in which they work or plan to work [10,16,17], training health professionals through a community placement programme plays a crucial role in eventual choice to work in rural areas [18].

There are several limitations to the study. The evaluation was carried out by staff of the college, most of whom were in favor of the community-based programme and were on the committee that administers the programme. This could have created some bias. Secondly, the questionnaire to first-year students was administered during their first meeting with the college administration, and some could have known about the community-based programme and how it involves having part of the training at rural health units. This could have influenced them to give only the views acceptable to the college faculty and administration. There we made no attempt to validate the responses from the in-depth interviews by presenting them to the group of the students. There were also no attempts to find whether continuing medical students, after exposure to rural health facilities during community-based training, had similar views or had their perceptions changed by the community placement.

## Conclusion

Many first year medical students at Makerere University have limited exposure to health facilities in rural areas and have concerns about eventually working in rural areas, which are related to personal factors, health system factors, socio-economic factors and prior exposure to rural health facilities. The decision to work in rural areas might be facilitated by exposure to rural practice during training, an understanding of rural needs and exposure to rural healthcare systems and their management role models. The context and nature of work environment opportunities it offer in terms of skills development, continuing professional development and opportunities for upgrading might influence the decision to apply for deployment in rural areas. Lastly, the welfare of medical personnel in terms of social amenities, support from family and friends, and a positive relationship with the community might influence recruitment or retention of medical workers in rural areas. Multiple factors influence working in rural areas, and community-based training is just a single component. Financial incentives need to be considered for individuals working in rural areas.

## Competing interests

AM heads the Community-based Education task force which champions the running and conduct of community-based education at the former Faculty of Medicine, currently College of Health Sciences. DKK, PS and JT are members of staff of the college, and serve as tutors and supervisors of students on community placements. NS is the former Dean of Faculty of Medicine and is currently Principal of the College of Health Sciences.

## Authors' contributions

DKK and JT conceived the study, and worked with PS, AM and NS to design the questionnaire. DKK collected and analyzed the data. All authors were involved in interpretation of the findings, contributed to editing the draft manuscripts and approved the final manuscript.

## Pre-publication history

The pre-publication history for this paper can be accessed here:

http://www.biomedcentral.com/1472-6920/10/47/prepub
